# Communication Abilities, Assessment Procedures, and Intervention Approaches in Rett Syndrome: A Narrative Review

**DOI:** 10.3390/brainsci15070753

**Published:** 2025-07-15

**Authors:** Louiza Voniati, Angelos Papadopoulos, Nafsika Ziavra, Dionysios Tafiadis

**Affiliations:** 1 Department of Health Sciences, Speech and Language Therapy, European University, Nicosia 22006, Cyprus; 2 Department of Speech and Language Therapy, School of Health Sciences, University of Ioannina, 45 500 Ioannina, Greece; angelos@uoi.gr (A.P.); nziavra@uoi.gr (N.Z.); tafiadis@uoi.gr (D.T.)

**Keywords:** Rett syndrome (RTT), communicative abilities, assessment, intervention, speech-pathology, narrative review

## Abstract

**Background/Objectives:** Rett syndrome (RTT) is a rare neurodevelopmental disorder that affects movement and communication skills primarily in females. This study aimed to synthesize the research from the last two decades regarding the verbal and nonverbal communication abilities, assessment procedures, and intervention approaches for individuals with RTT. **Methods:** A structured literature search was conducted using the Embase, Scopus, and PubMed databases. Fifty-seven studies were selected and analyzed based on inclusion criteria. The data were categorized into four domains (verbal communication skills, nonverbal communication skills, assessment procedures, and intervention approaches). **Results:** The findings indicated a wide variety of communicative behaviors across the RTT population, including prelinguistic signals, regression in verbal output, and preserved nonverbal communicative intent. Moreover, the results highlighted the importance of tailored assessments (Inventory of Potential Communicative Acts, eye tracking tools, and Augmentative and Alternative Communication) to facilitate functional communication. The individualized intervention approaches were found to be the most effective in improving communicative participation. **Conclusions:** The current review provides an overview of the current evidence with an emphasis on the need for personalized and evidence-based clinical practices. Additionally, it provided guidance for professionals, clinicians, and researchers seeking to improve the quality of life for individuals with RTT.

## 1. Introduction

Rett syndrome (RTT) is a rare neurodevelopmental disorder caused by a genetically based mutation of the methyl-CpG-binding protein 2 (MECP2) gene on the X-chromosome that occurs almost entirely in females in early childhood [1,2,3]. Mutations in the gene encoding MECP2 are related to rare familial cases of RTT and typical RTT [2]. By employing a variety of advanced mutation detection tests, it is possible to identify mutations in the MECP2 gene in around 95 to 97% of patients who exhibit typical symptoms of RTT [3,4,5,6]. However, even with the most effective techniques, 3 to 5% of patients who meet all the clinical criteria for RTT are found to be missing a known mutation in the MECP2 gene. This suggests that a mutation in this gene is unnecessary for diagnosing typical RTT [3,4]. Of the diagnosed cases, 88.4% showed a positive mutation of the MECP2 gene upon testing. In unusual cases, recognized mutations in the MECP2 gene are present in only 50 to 70% of affected individuals. Additionally, MECP2 gene mutations have been identified in individuals who do not display the clinical features associated with RTT [3,4,5,6].

### 1.1. Diagnostic Criteria for Rett Syndrome

In 2010, Neul et al. [3] updated the 2002 diagnostic clinical criteria for typical Rett syndrome to enhance clarity and streamline the diagnostic process. The revised criteria encompassed the key criterion of regression, along with four key signs important for diagnosing typical Rett syndrome (RTT). The characteristic symptoms of typical RTT include a decline in purposeful hand movements and verbal communication, accompanied by the development of atypical walking patterns and repetitive hand stereotypies [3,7]. The median age for diagnosing classic Rett syndrome is typically 3 years, with a diagnostic range spanning from 2 to 4 years. Andreas Rett reported that all children were healthy until 9 months but experienced some feeding issues. In addition, reports revealed the first neurological dysfunctions at 8–10 months with stagnated development and diminished social interest by 15 months. According to a review study [8] detailing the acquired speech and language and socio-communicative abilities of females with RTT before the onset of regression, the authors reported that all infants who were later diagnosed with RTT engaged in eye contact before regression. However, the examination of videos from children during the initial 6 months of their lives revealed that many infants displayed frozen, developmentally unusual smiles at 2.5 months and again at 6 months of age [8]. The authors of the review noted that during the regression phase, children exhibited reduced interest in objects and social interactions and did not respond when their name was called, despite sustaining eye contact [8]. For typically developing infants, the act of producing soft, prelinguistic vocal sounds progressively declines, eventually ceasing between 6 and 10 months of age, and is replaced by the early babbling stage. The early stage of babbling is then followed by the production of more complex syllables and babbling noises, including a mixture of different sounds [8]. After a phase of development that appeared typical but showed subtle impairments, recent evaluations of receptive language skills indicate that some individuals with Rett syndrome may exhibit cognitive abilities within the below-average or mild cognitive impairment range [9]. However, as late infancy progresses, affected individuals can experience a decline in functioning, leading to severe intellectual disability and serious neurological complications [10,11].

Between the ages of 5 and 18 months, individuals with Rett syndrome may experience either progressive deterioration or a relatively slow improvement in motor and adaptive functioning, affecting cognitive and communicative abilities [9]. Additionally, they frequently exhibit impaired communication skills [12,13], sensory processing challenges [14], stereotyped hand movements [15], unexplained respiratory irregularities such as hyperventilation [8], seizures [16], scoliosis [17], and motor disorders like spasticity [18], which may eventually lead to wheelchair dependence [19]. Reduced ambulation may lead to dystonia, which can result in foot and hand deformities [20].

As RTT progresses, affected individuals may develop commonly co-occurring conditions, including episodes resembling panic attacks, bruxism, ataxia and apraxia, tremors, and acquired microcephaly. People with RTT generally live into adulthood, with an average life expectancy of around 50 years, underscoring the long-term need for interdisciplinary care and tailored communication interventions throughout the lifespan. In this context, understanding the evolving nature of communication impairments becomes critical to enhancing the quality of life for both individuals with RTT and their caregivers.

### 1.2. Rett Syndrome and Communication Characteristics

One of the key diagnostic criteria suggesting the possibility of RTT in a child is deterioration and significant weaknesses in their communicative repertoire [12,13,21]. Several studies have indicated that individuals with RTT often experience a range of prelinguistic behaviors needed for communicative purposes (basic oral, verbal, and gestural expressions and nonverbal communication with signing, gestures, nods, sounds, touch, and eye interaction) [8,21], but their speech and language development conspicuously include at most only a few single words [17,22,23]. However, across the literature, there is notable variability in how communication abilities are defined, measured, and interpreted. Studies differ in their use of terminology, assessment procedures, and outcome reporting, which contributes to a fragmented and inconsistent knowledge base on communication in RTT. This makes it difficult for researchers and clinicians to draw definitive conclusions or compare findings across studies.

In addition, autistic behaviors observed in individuals with RTT can implicate the ability to participate in joint social interaction, impaired verbal and nonverbal shared communication, and limited imagination with repetitive and fixed behaviors [24,25]. The presence of symptoms that overlap with autism spectrum disorders, such as stereotyped behaviors, impaired social reciprocity, and restricted communication, can obscure the accurate diagnosis and categorization of communicative deficits specific to RTT. This overlap complicates both clinical decision-making and the selection of appropriate assessment instruments, which are often not normed for RTT-specific profiles. Moreover, the potential for misdiagnosis or diagnostic overshadowing may result in interventions that are not adequately tailored to the nuanced needs of the individual. These communication deficits influence the quality of life of an individual with RTT [26]. Thus, individuals with RTT cannot express their feelings or personal needs to live a quality life [26]. Effective communication skills are essential for achieving a high quality of life, as they play a crucial role in building relationships, shaping identity, fostering personal development, and engaging socially.

Communication difficulties also place a considerable emotional and logistical burden on caregivers, who are often responsible for interpreting subtle cues and advocating for appropriate services. Additionally, cultural and linguistic contexts may influence how symptoms are interpreted and the accessibility of augmentative strategies, thereby limiting the generalizability of some findings to diverse populations. Diverse cultural attitudes toward disability, gender, and nonverbal expression may influence how symptoms are identified, reported, and addressed. For example, in some cultural settings, limited verbal output might be perceived as shyness or developmental delay rather than a potential marker of a neurodevelopmental disorder, thereby delaying diagnosis and intervention [27].

Access to communication assessments and interventions—especially those involving advanced technologies like AAC or eye-tracking—also varies significantly across geographical regions due to disparities in healthcare infrastructure, clinician training, funding, and the language availability of tools. While advances in AAC technology, such as eye-tracking systems, tablet-based applications, and even neuroimaging tools, have opened new pathways to support communication, these resources remain underutilized or insufficiently addressed in many studies. Their integration into assessment and intervention models warrants further exploration [28].

Numerous studies and reviews have explored the communication abilities of children with RTT [12,13,29,30]. Additionally, some research has concentrated solely on assessment methods for communication abilities [8,31], while others have focused on therapeutic strategies [12,13,32,33]. Yet, despite this growing body of literature, there remains no unified synthesis that integrates communication characteristics, assessment procedures, and evidence-based intervention strategies into a coherent framework. This critical gap underscores the urgent need for a comprehensive, up-to-date narrative review that not only summarizes findings but also highlights practical considerations for clinical application.

Although numerous studies have been conducted, the past two decades have lacked a comprehensive review that systematically consolidates all available information on the communication difficulties experienced by individuals with Rett syndrome. Such a review is crucial for improving the identification and management of these challenges. There is a critical need for research to consolidate all this information, enabling SLPs and other professionals to gain an extensive understanding of the communication abilities of individuals with RTT, along with detailed knowledge of assessment and intervention methods [26]. By consolidating existing knowledge, SLPs and other professionals would gain a more comprehensive perspective on the unique communication challenges faced by this population. Additionally, the research would enhance the precision and effectiveness of assessment and intervention strategies, allowing clinicians to tailor their approaches based on a deeper, evidence-based understanding. This, in turn, could lead to improved interaction opportunities, greater social engagement, and ultimately a higher quality of life for individuals with RTT [12,13,22,29].

### 1.3. Purpose of the Present Review

In response to the critical need for a comprehensive understanding of the communication abilities of individuals with Rett syndrome, along with effective assessment and intervention methods, a narrative review protocol was chosen. This approach enables the systematic integration of extensive data on both verbal and nonverbal communication skills, while also providing valuable insights into evidence-based strategies for improving communication outcomes in individuals with RTT.

An integrative narrative review offers a unique advantage by bridging diverse research methodologies and translating complex evidence into accessible guidance for clinical and educational settings. This synthesis not only maps the landscape of current knowledge but also identifies areas for innovation in diagnostic practices and therapeutic support [34]. Moreover, this type of narrative review critically evaluates and integrates publications from evidence-based studies employing diverse methodologies, ensuring a comprehensive synthesis of existing research [35]. By identifying patterns, inconsistencies, and emerging trends in the literature, this review aims to inform future research priorities and enhance multidisciplinary collaboration for improved communication support across the lifespan of affected individuals.

This narrative review was conducted to address the research question: What are the communication abilities of individuals with Rett syndrome, and how can professionals effectively assess their communication repertoire and implement appropriate intervention strategies? The primary objective of the review was to identify and synthesize existing research on communication skills, assessment methodologies, and intervention approaches for individuals with RTT. Specific objectives included:

1. Categorize the communication abilities of individuals with RTT participating in research into verbal and nonverbal skills.
2. Indicate possible speech and language assessment procedures for identifying the communication abilities of research participants and clinical clients with RTT.
3. Identify potential intervention approaches for SLPs and other professionals involved in assessing, planning, and implementing interventions to address communication deficits in individuals with RTT.

## 2. Materials and Methods

### 2.1. Search Strategy

The present review adhered to the structured 12-step approach outlined by Kable et al. (2012) [36] to ensure a systematic and comprehensive evaluation of the relevant literature. Each step was carefully implemented, beginning with the formulation of the research question, which aimed to examine the communication abilities of individuals with Rett syndrome, as well as the assessment and intervention strategies employed by professionals.

A thorough literature search was conducted in June 2024 using three well-established electronic databases: Embase, Scopus, and PubMed. We used the terms and the Boolean operators as below: (“Rett Syndrome” OR “Rett disorder” OR “MECP2”) AND (“communication” OR “communicative abilities” OR “language” OR “speech” OR “verbal” OR “nonverbal”) AND (“assessment” OR “evaluation” OR “diagnosis” OR “screening”) AND (“intervention” OR “therapy” OR “treatment” OR “rehabilitation” OR “speech therapy” OR “language intervention”). Regarding the filters, to ensure the inclusion of high-quality and relevant studies, clear search parameters were applied, focusing on published articles from the last two decades (2004–2024). The search terms, as described before, allowed for a targeted and comprehensive exploration of available research. Moreover, backward citation tracking was performed to identify additional relevant studies beyond those retrieved directly from the databases. The Mendeley Reference Manager was used to help us organize citations and remove duplicate studies. The data were extracted using an Excel matrix for (a) authors, (b) sample, (c) sample’s age, (d) purpose of the study, (e) study design, (f) communication skills, (g) assessment of communication skills, and (h) intervention of communication skills. To reduce bias and increase methodological transparency regarding interrater agreement, two independent researchers conducted the screening process. In the event of any disagreement, a consensus was reached.

The retrieved studies were systematically screened based on predefined inclusion and exclusion criteria, ensuring relevance and reliability. Titles and abstracts were reviewed to verify their alignment with the objectives of the present study. To synthesize existing knowledge effectively, the selected articles were categorized into four primary domains: (a) verbal competencies, (b) nonverbal competencies, (c) speech and language assessment, and (d) intervention strategies. This classification facilitated a structured analysis of communication characteristics in individuals with Rett syndrome.

Furthermore, an expert consensus on the current context and potential future developments in the field was examined across each of these four areas. The findings from this process were systematically documented, providing a synthesized overview of key insights. Additionally, the study process is visually represented in the following flow diagram, illustrating the methodological framework applied throughout the review.

By adhering to these guidelines, the present review ensured a robust methodological foundation, maintaining reliability and relevance in its findings. The inclusion of specific search parameters, keywords, and time frames enabled a comprehensive synthesis of the most pertinent literature on communication abilities in individuals with Rett syndrome, ultimately supporting the development of evidence-based assessment and intervention strategies.

### 2.2. Study Selection Criteria

**Figure 1:**
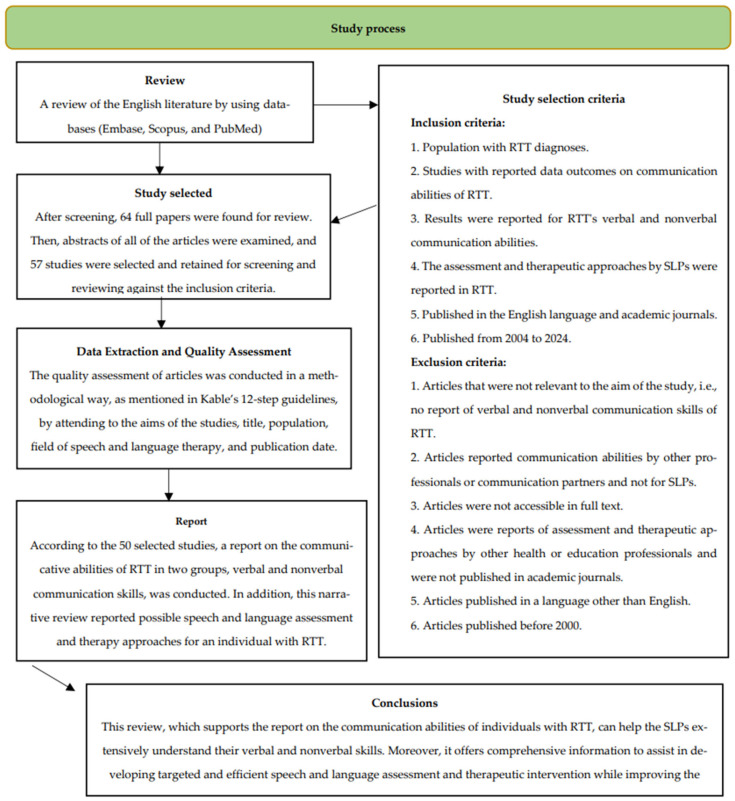
Flow diagram of the description of the study process.

A comprehensive literature search was carried out utilizing major academic databases, including Embase, Scopus, and PubMed, to ensure a thorough evaluation of relevant studies. Given the narrative nature of this review, PRISMA guidelines were not applied, as the focus was on synthesizing the existing literature rather than conducting a systematic review. Research articles were screened, duplicates were disregarded, and 64 full papers were found for review. Then, abstracts of all articles were examined, and 57 studies were selected and retained for screening and reviewing against the inclusion criteria (see Figure 1). Inclusion criteria further focused on recognizing the adequacy of the information presented in the research. Two [2] independent researchers evaluated the eligibility of the studies. Following this assessment, the full texts of the selected studies were retrieved for further analysis. The two researchers worked collaboratively to ensure that all studies met the established eligibility criteria, resolving any potential discrepancies to maintain consistency in the review process. The selected papers met the established criteria, offering relevant information that aligned with the objectives of the review. Additionally, the chronological distribution of the included studies was documented to ensure a comprehensive assessment of the evolving research landscape.

The inclusion criteria were as follows:

1. Population with RTT diagnosis;
2. All the types of studies with reported data outcomes on the communication abilities of RTT;
3. The findings focused exclusively on the verbal and nonverbal communication abilities of individuals with Rett syndrome, without imposing restrictions based on participant characteristics;
4. The assessment and intervention approaches implemented by SLPs and other healthcare professionals in individuals with RTT were documented;
5. Published in peer-reviewed academic journals available in the English language;
6. They were published from 2004 to 2024.

This review encompasses studies that present data on the verbal and nonverbal communication abilities of individuals with Rett syndrome, along with approaches for their assessment and intervention.

The exclusion criteria were as follows:

1. Articles irrelevant to the study’s aim that did not report the verbal and nonverbal communication skills of individuals with RTT;
2. Articles reported communication abilities assessed by other non-SLP professionals or communication partners;
3. Articles were inaccessible to the authors in full text;
4. Articles reported assessment and intervention approaches by other health or education professionals or were not published in peer-reviewed journals;
5. Articles were published in a language other than English;
6. Articles published before 2004.

### 2.3. Data Extraction and Quality Assessment

As previously outlined in this review, the study adhered to a structured 12-step framework to document the search strategy before conducting a critical analysis and synthesis of the retrieved literature. Following Kable’s 12-step guidelines (2012) [36], the review systematically examined the studies’ objectives, titles, target populations, relevance to speech and language therapy, and publication dates. Currently, there are no universally recognized guidelines for formulating narrative reviews, which present two primary limitations: (a) the inherent biases related to assumptions, planning, selection, and evaluation are often not explicitly identified, and (b) the lack of reproducibility in narrative studies. However, to enhance the methodological rigor and ensure a higher quality standard in this review, the researchers employed the Scale for the Assessment of Narrative Review Articles (SANRA) as a guiding framework during manuscript preparation [37,38].

The Scale for the Assessment of Narrative Review Articles (SANRA) was developed between 2010 and 2017 by a team of editors as a concise and practical tool for evaluating the quality of narrative review articles. It serves as a guideline for editors, reviewers, and readers in assessing both the rigor of academic papers and the effectiveness of authors in structuring narrative reviews. In 2014, the authors refined SANRA to enhance its clarity and reliability, streamlining the wording of its items and eliminating a criterion related to manuscript writing and accessibility due to inconsistent ratings [37,38].

The revised SANRA scale consists of six criteria, each rated on a scale from 0 (low quality) to 2 (high quality), with a maximum possible score of 12. The six assessment components include (1) the explanation of the review’s significance, (2) the articulation of the review’s objectives, (3) the description of the literature search process, (4) the accuracy and adequacy of referencing, (5) the strength of the scientific reasoning, and (6) the presentation of relevant endpoint data [37].

In the present review, SANRA was employed as part of the self-assessment process for the current study’s data extraction and quality assessment to ensure methodological rigor and enhance the reliability of the findings. The review was systematically assessed according to the SANRA criteria, allowing for a structured evaluation of the comprehensiveness, relevance, and scientific validity of the selected literature. By integrating SANRA into the quality assessment framework, this review aimed to maintain high academic standards while synthesizing the existing knowledge related to communication abilities, assessment, and intervention strategies for RTT. Appendix B presents the SANRA evaluation of the current study.

## 3. Results

Based on the analysis of 57 selected studies, the communicative abilities of individuals with Rett syndrome have been examined within two primary domains: verbal and nonverbal communication skills. Furthermore, a range of speech and language assessment methods and intervention strategies have been employed across research studies to evaluate and support the communication development of individuals with RTT (see Table A1 in the Appendix A).

### 3.1. Sample Characteristics

To reduce potential sample overlap in commonly conducted research, the sample characteristics were systematically classified according to study type. Specifically, the studies were organized into three distinct categories: empirical research studies, literature review studies, and studies in which the sample size was not explicitly reported. This classification ensured a structured approach to data organization, facilitating a clearer understanding of the participant demographics and methodological variations across the included studies. By distinguishing between these categories, the review aimed to enhance the accuracy of data interpretation, prevent redundancy, and provide a more comprehensive synthesis of communication-related findings in individuals with Rett syndrome. The narrative review incorporated 30 selected research studies, collectively analyzing data from 1052 individuals diagnosed with RTT.

Additionally, six of these research studies examined the perspectives and experiences of 1764 parents, caregivers, SLPs, and other professionals working with individuals with RTT. Further, four of the selected research studies included comparative analyses between 93 individuals with RTT and 96 typically developing females, contributing to a broader understanding of communication differences in this population. In addition to empirical research, seven of the 57 selected papers were literature reviews focused on individuals with RTT, increasing the total number of examined studies to 187. Lastly, the remaining ten studies did not specify the exact sample size, though they provided relevant insights into communication assessment and intervention strategies for individuals with RTT.

### 3.2. Verbal Communication Abilities

Based on the extracted data from the included studies examining verbal communication abilities, individuals with RTT demonstrated multiple deficits. Attention-related limitations appeared to hinder their learning capacity, as supported by findings in the literature [15,19,39]. The findings indicate that enhancing cognitive functions in individuals with RTT may contribute to more purposeful and effective communication [40]. However, this is often constrained by motor impairments, such as difficulties in coordinating hand movements and oral motor control required for speech production [17,28], which were observed in several of the reviewed studies.

Although there is a notable accomplishment in speech and language milestones, such as cooing, babbling, first words, and even word combinations, during the first two years of life, longitudinal analyses indicate a subsequent regression characterized by reduced verbal output and diminished social engagement in RTT [8,21,31,39,41]. This developmental regression has been linked to a deterioration in speech, language, and overall communicative abilities, alongside a reduced inclination for social interaction and impairments in purposeful hand use [8,34,42].

Several studies documented the emergence of idiosyncratic verbalizations and limited topic maintenance despite gradual expansion in articulation, phonological awareness, and mental vocabulary [31,41] Behaviors such as finger-pointing [43] and repetition of known common words, along with the refusal to follow directions or respond to their name, can indicate a decrease in their mental lexicon development [8,15,22]. They can often lose proto-conventional words but build on their passive lexicon skills [8,21,33].

Additionally, a significant phonological deficiency has been detected, along with poor precision of the articulation mode, thus inhibiting overall speech intelligibility [44,45]. Verbal abilities are further hindered by morphosyntactic and pragmatic errors and disfluency, possibly resulting from immediate echolalia or questioning [22].

Multiple studies also reported a progressive decline in vocalization ability, even when expiratory airflow was present, coinciding with increased motor stereotypies—such as repetitive hand-to-mouth movements—which may serve as diagnostic indicators for RTT and its variants [8,46,47]. Individuals with RTT who can no longer use speech to communicate also display inconsistent imitation and social skills [9]. Despite this, observational studies reveal that many maintain expressive language at a pre-intentional level, evidenced by the use of specific nonverbal cues with potential communicative intent. These include vocalizations [9,35,48], laughter and crying [9], facial expressions [49,50], touch, and gazing [51]. The inability of individuals with Rett syndrome to regain speech skills can be primarily attributed to two key factors: the presence of severe dyspraxia, which significantly impairs motor planning for speech production, and, in some cases, cognitive abilities that may fall within the below-average to mild range [52].

### 3.3. Non Verbal Communication Abilities

The disengagement of verbal communication and the regression of language abilities often occur after reaching Stage I. At the same time, some children display some form of nonverbal prelinguistic communication, which is also seen in later stages [45]. Empirical observations confirm that many individuals with RTT continue to use nonverbal channels well into later stages of development. Several studies investigating developmental trajectories in typically developing infants demonstrate that early preverbal communication emerges through shared social engagement [8,41]. The child uses communicative acts such as pointing or reaching out to show or request objects [8,53,54,55]. The action of more than two persons directing their attention to a common drive of interest—‘joint attention’—is an important part of this informative communicative process [20,55,56]. Making eye contact and following the gaze of others while attending to their focus of interest (eye-direction detection’) are behaviors necessary for developing joint attention. Such behaviors also play a vital role in identifying movements as a means of intentional communication [21].

Nonverbal communication can be exhibited through eye gaze/eye contact to request a non-expressive interaction [34,46,49]. Caregiver and clinician reports highlight the use of body positioning, gestures, and affective signals such as frowning or withdrawing to express preferences or discomfort [8,25,48]. Eye pointing is used clearly for communicative intentions and gestural attention [52,57,58,59].

Research examining language comprehension in individuals with RTT, including both classical and forme fruste (a partial symptom of the syndrome) presentations, has indicated that they primarily recognize their names, as well as key words or simple sentences. These identifications are largely influenced by visual or contextual cues [29,53].

However, receptive abilities are difficult to assess consistently, as moderate to severe motor impairments often limit active response options. Many studies, therefore, evaluated comprehension relative to estimated mental age or through proxy measures, such as gaze preference or behavioral responses [45]. These challenges underscore the need for individualized, multimodal approaches to assessing and interpreting receptive communication in RTT.

### 3.4. Speech and Language Assessment Procedure

Considering the need for accurate assessment, numerous sources of information, such as medical records, parent and staff reports, video recordings, and clinical observations, were used to explain the participants’ developmental history and current functioning [43]. Standardized methods are often found to be limited, largely due to the lack of assessment tools specifically tailored to the unique communicative and cognitive profiles of individuals with RTT [60].

To assess the potential communicative forms and functions of existing prelinguistic behaviors in individuals with severe disabilities such as RTT, the Inventory of Potential Communicative Acts (IPCA) was used in several studies. These studies reported that, despite extremely limited behavioral repertoires—such as eye gazing or simple body movements—children with RTT exhibited consistent patterns interpreted by caregivers and educators as communicative acts [30,47].

The communicative functions inferred from these behaviors typically included acknowledgment, initiating dialogue, requesting objects, and expressing rejection [29]. However, these interpretations were based primarily on informant-reported responses via the IPCA, and some studies cautioned that clinical observation alone did not always verify these functions conclusively, reflecting the need for complementary assessment methods to validate communicative intent [61].

Receptive language abilities were assessed in multiple studies using both technology-based and traditional instruments. These included eye-tracking technology (ETT) and standardized tools such as the Peabody Picture Vocabulary Test (PPVT) [62]. The Assessment of Visual Attention in Interaction (AVAI) tool, developed to assess visual attention operationalized as concentrated gazes at the communication partner, an object, and a symbol set, might also be beneficial throughout the receptive and expressive language skills assessment procedure. The AVAI tool enables the assessment of visual attention in more realistic interactions with communication partners [13].

Other tools for the identification of the girls’ communication levels include: the Early Language Milestones (ELM) Scale, the Vineland Adaptive Behavior Scales (VABSs) [53,63,64,65], structured descriptive assessment (SDA) [66], and the parent–interview portions of the Sequenced Inventory of Communicative Development (SICD), along with medical records [30]. These tools were often used in conjunction with clinical records to provide a more holistic picture of communicative functioning, motor skills, and associated medical conditions.

Some studies also described scoring approaches to quantify total communication abilities—for example, by assigning weighted values to behaviors such as eye pointing, cause/effect signaling, and the ability to make choices—providing a structured means of summarizing observed communicative intent [58,61].

The caregiver-completed Rett Syndrome behavior Questionnaire (RSBQ) is one of the most extensively used effectiveness measures in the clinical evaluation of RTT, due to its specificity for essential RTT symptoms, including eye gazing and expressionless facial features [57]. Moreover, the Rett Assessment Rating Scale (RARS) enables a complete and reliable evaluation of individuals with RTT by individually analyzing and rating key features, including the cognitive area, which encompasses communication skills [49,55]. Another useful tool is the Responsive Augmentative and Alternative Communication Style Scale (RAACS), which was created to measure parents’ communication styles with children who have difficulties with communication [46]. Furthermore, for the Communication and Symbolic Behavior Scales Developmental Profile Infant–Toddler Checklist (CSBS-DP-IT) [43,67], the social composite score generated from the CSBS-DP which was designed to measure communication and social interaction abilities in young children (ages 12 to 24 months), could also be considering in assessment of affected individuals. It may also be used with older children with developmental delays, including those with RTT [67]. RTT-specific clinician rating scales related to discrete aspects of communication (RTT-COMC) are a clinician-completed assessment of the individual’s ability to communicate their choices or preferences, including the use of nonverbal means such as eye contact or gestures, using an eight-point Likert scale [67,68].

Across the studies included, findings suggest that a subset of individuals with RTT retain communicative potential identifiable through structured assessment of prelinguistic behaviors. These findings reinforce the importance of early, individualized, and multimodal communication assessments. They also support therapeutic approaches that actively build upon observable communicative intent rather than relying solely on verbal performance as a metric of ability [34].

### 3.5. Speech and Language Intervention Approaches

Based on the assessment findings, four distinct modes of communication have been identified as key components of the intervention process. These include speech or vocalization, gestures, graphic symbols and/or written language, and electronic or computer-based systems such as Assistive Augmentative Communication (AAC). The reviewed studies indicate that these modalities support various levels of communicative engagement, particularly among individuals with limited verbal output. AAC methods can involve speech-generating devices, computers with speech output, graphic symbols [13], and printed vocabulary systems.

Most individuals with RTT demonstrate significant limitations in expressive language, while their receptive comprehension is often relatively preserved [33,60]. Studies have shown that integrating AAC strategies can help bridge this expressive–receptive discrepancy, enabling greater participation in communication exchanges. The role of the SLP is to identify appropriate modalities and tailor intervention plans accordingly. AAC devices are divided into high, mid, and low-tech. High-tech AAC encompasses speech-output systems such as touch-screen iPads or tablets that are manually activated, as well as Tobii devices that operate through eye gaze. These tools have demonstrated effectiveness in controlled settings for facilitating sentence construction and initiating communication. High-tech systems use a digital or synthesized voice, enable computer interfacing, and have features like text-to-speech and sentence creation. Mid-tech AAC (e.g., microswitches) lacks some speech-output technologies. There are no electronics in low-tech AAC. A few instances are choice boards and communication books [28,66]. Among specific approaches reported in intervention studies, ‘mind training’—where AAC is used to request reinforcers with single-word prompts—has been cited as effective when implemented through graduated cueing and prompt-fading procedures [69]. In addition to AAC-based techniques, some studies have explored the use of Transcranial Direct Current Stimulation (tDCS) as a therapeutic modality. While research is preliminary, positive outcomes were noted in speech production and comprehension, suggesting potential as an adjunct to behavioral interventions [64]. Other methods for intervention are to teach speech or communicative intent through communicative gestures (for example, crossing arms, eye gaze, and pointing) [65,66], graphic symbols [13], or using a communication board while enabling the individuals with RTT to make choices and interact [43]. The reviewed literature emphasizes that the use of these strategies should be grounded in individualized assessment results and responsive to the child’s developmental stage.

Numerous researchers vouch for the need for intensive, continued treatment to improve the long-term outcome in syndromes, and specifically in neurodevelopmental disorders. Intervention planning should, on the one hand, deal with the present functional and medical actuality, but it should also focus on the long-term prognosis of the potential of an individual with RTT [64]. This dual focus requires alignment between therapeutic goals, educational programming, residential supports, and future vocational considerations [30,34].

A successful intervention program must also include the adequate participation and cooperation of the individual’s family. Empirical studies suggest that individualized, multi-domain intervention programs—targeting communication, cognitive functioning, and motor abilities—yield the most beneficial outcomes [67,70]. Regardless of the individual’s age, treatment planning should establish precise learning opportunities and generalization of what is learned [71]. Developing an effective IEP requires goal-oriented planning that incorporates communication-specific objectives, AAC strategies, and partner training [72]. Furthermore, to establish the IEP, a structured, intervention-oriented procedure is necessary. It is suggested that the communicative functions and partner strategies be targeted in general communication intervention and communication aids (CAs) [13,33,34,46,72,73].

The communication skills targeted for intervention were coded into pragmatic functions based on the classification system described by Sigafoos, Arthur-Kelly, and Butterfield [74]: imitative speech, joint attention, turn-taking, asking, requesting access to preferred stimuli (e.g., food, drinks, toys, or songs), requesting social interaction/topic initiation, naming objects or commenting, narrating, following instructions, and receptive language. Partner strategies that support these functions involve: modeling communication behaviors, allowing extra response time, recognizing and expanding on interests, regulating stimulation, gaining attention, and respecting communicative intent through supported choice-making [9,55].

When conventional approaches yield limited results, identifying an effective Communication Aid (CA) can guide the next phase of intervention. Reported examples include single-message speech-generating devices [69,75], visual communication charts [44,46,60], picture exchange cards [54], and both low-tech (books, boards) and high-tech (eye-gaze tablets, iPads) systems [57,69,74]. Communication passports, a PECS (Picture Exchange Communication System), talking mats, multi-message AAC systems, and custom apps were also identified in the literature as tools that support individual needs and enhance communication independence [15,75].

## 4. Discussion

This review aimed to provide further knowledge and report on the communicative intent of individuals with RTT regarding communication abilities, assessment procedures, and intervention approaches. The study’s strength lies in its synthesis of findings from the past two decades, offering a comprehensive understanding of communication challenges and support strategies that can inform clinical practice and enhance multidisciplinary approaches.

### 4.1. Communication Abilities of Individuals with RTT (Verbal and Nonverbal)

In terms of communication abilities in individuals with RTT, studies by Cianfaglione et al. [45], Roche et al. [15], and Townend et al. [19] indicate that their verbal communication skills are closely tied to their cognitive abilities, which are difficult to evaluate. Fabio et al. [20] and Elefant and Wigram [39] agreed with previous researchers. They noted that improving cognitive abilities can help in deliberate communication. Still, these skills can be misunderstood due to their physical limitations in coordinating hand movements and the oral motor skills required to produce speech. All the researchers who analyzed RTT’s verbal communication skills noted that, gradually, vocalization skills decreased, and hand-to-mouth stereotypes increased, as individuals with RTT could no longer use speech to communicate and showed inconsistencies in imitation and social skills [8,61].

In addition, several studies concluded that verbal communication disengagement and language delay [3,17] typically occur after Stage I, with the children displaying nonverbal prelinguistic communication at later stages [8,41,45,53,54]. As it was indicated, individuals with RTT can engage in nonverbal dialogue through bodily expressions; their ability to convey engagement, reluctance, and displeasure is often manifested through gestures and other nonverbal cues, serving as an essential means of interaction and expression [21,56,65]. However, it is essential to note that many of these findings stem from studies with small sample sizes and heterogeneous RTT phenotypes, which limit generalizability. Additionally, much of the data rely on caregiver reports, which, while valuable, may introduce subjectivity or bias. These methodological limitations should be considered when interpreting conclusions.

Longitudinal research on communication development in RTT remains limited but essential. Understanding how verbal and nonverbal skills evolve across life stages—from early regression to adulthood—could guide more adaptive, age-appropriate interventions and monitoring strategies. These findings regarding the communication abilities of individuals with Rett syndrome, encompassing both verbal and nonverbal aspects, offer significant insights into how affected individuals express themselves. The existing literature emphasizes substantial variability in communication skills, highlighting differences in expressive capabilities and receptive processing.

Furthermore, the findings highlight the multifaceted challenges that individuals with RTT face in conveying their thoughts, emotions, and needs. Factors such as motor impairments, cognitive limitations, and speech-related deficits contribute to the complexity of their communication difficulties. Understanding these variations is crucial for developing effective intervention strategies, including tailored speech-language therapies and AAC systems [66,69]. Additionally, cultural, linguistic, and socioeconomic factors may significantly influence the interpretation of communicative behaviors, access to intervention tools (such as AAC devices), and caregiver engagement. These contextual influences are often underreported and should be addressed in future, more inclusive research.

### 4.2. Speech and Language Assessment Procedures

To accurately evaluate RTT’s verbal and nonverbal communication skills, an assessment of their developmental history and current functioning in persons with severe impairments, such as RTT, was necessary [47,60]. The IPCA was used to examine the possible communication forms and roles of current prelinguistic behaviors [42,60]. As an interview protocol, the IPCA aimed to identify the communicative responses of people with a developmental disability [76]. A recent narrative review discussed the findings of numerous studies that support the positive effects on the reliability and validity of the IPCA, as well as its utility for communication acts, profiles, and interventions for individuals with developmental disabilities [77]. Other research has found that the ELM Scale, VABSs, and the parent–interview sections of the SICD are often utilized during RTT communication evaluation [52]. The ELM scale [78] was used as a brief screening (parental/caregiver report, examiner observation, and direct testing) of the language abilities of children under the age of 3 years (how understandable the child’s speech is). This assessment consists of three sections: auditory expressive, auditory receptive, and visual [78,79]. Furthermore, as indicated in the literature [71,72,73,74,75,76,77,78,79,80,81,82,83], it was crucial to use a VABS as a standardized assessment tool that utilizes semi-structured interviews to measure adaptive behavior and assist in the diagnosis of developmental disabilities. Regarding the utilization of the SICD [84], this tool assesses the language development of children by evaluating expressive communication (including imitation and generation of sounds and words) and comprehension of commands [84,85].

Despite the utility of these instruments, many were not designed initially or normed for the RTT population. As such, their sensitivity and specificity in capturing nuanced communicative intent, particularly nonverbal behaviors, are limited. This further underscores the need for RTT-specific assessment tools that encompass the full range of communicative capacities. Emerging technologies such as eye-tracking systems, brain–computer interfaces (BCIs), and AI-assisted AAC platforms offer promising advances in assessing and supporting communication in nonverbal or minimally verbal individuals with RTT.

These approaches aim to identify strengths and limitations in verbal and nonverbal expression, facilitate alternative communication methods, and enhance overall interaction abilities. The findings contribute to a broader understanding of evidence-based practices for assessing communication in individuals with RTT, informing future clinical applications and research directions.

### 4.3. Intervention Approaches for SLPs and Other Professionals

Some researchers have provided a precise picture of communication forms and goals in individuals with RTT, suggesting that some children may retain communicative skills detected by prelinguistic behavior [30,34].

Five different communication forms are inferred from assessment results: speech, gesture, graphic symbols, written lexicon, and electronic or computer-based systems (AAC) [15,86]. Thus, interventions to enhance speech or communicative intent are attempted through gestures, graphic symbols, or a communication board. Intervention planning should consider both the present functional and medical actuality and the long-term prognosis of a child’s potential with the adequate involvement of the family [33,75].

However, intervention planning must extend beyond speech–language pathology. The coordination of care with occupational therapists, psychologists, educators, and neurologists is vital to addressing the multifactorial needs of individuals with RTT. Multidisciplinary collaboration ensures that motor, cognitive, emotional, and sensory components of communication are not overlooked. An IEP for individuals with RTT should be directed by identifying intervention methods used to assess and evaluate communication skills and the overall aims and estimated outcome of a general communication intervention [30,34,64]. Communication skills targeted for intervention are coded into pragmatic functions based on the classification system described by Sigafoos, Arthur-Kelly, and Butterfield. Partner communication strategies’ target skills are imitative speech, joint attention, turn-taking, asking, requesting access to preferred stimuli, requesting social interaction, naming objects or commenting, narrating, following instructions, and receptive language [70]. Recognizing adequate and efficient communication aids facilitate further intervention when other means have been unsuccessful [44,53,73,74].

While families are acknowledged in intervention planning, their ongoing role in training, emotional support, and implementation is equally crucial. Sustained caregiver engagement—bolstered through accessible resources and coaching—can greatly influence outcomes and reinforce skills across home and community environments.

Ethical considerations must also be integrated into communication intervention, including the individual’s right to express themselves meaningfully, the need for informed consent in research or clinical settings involving nonverbal populations, and broader advocacy for inclusive access to communication support tools and services.

Although past research and case studies have provided us with a wealth of data and facts on the communication abilities of individuals with RTT, many of these findings are based on small samples, including diverse RTT phenotypes, and frequently rely on caregiver-reported outcomes. These methodological limitations must be acknowledged when interpreting communication profiles and assessing intervention effectiveness [15,44,45]. This presents a critical opportunity to conduct an in-depth examination of the existing intervention methodologies and explore innovative approaches for enhancing support for individuals with RTT who experience challenges with both verbal and nonverbal communication. Notably, longitudinal and developmental perspectives are underrepresented in the current research. The evolution of communication abilities across different life stages—from early childhood to adulthood—warrants a more systematic study to tailor interventions to the changing needs of individuals over time.

The ongoing refinement of current practice techniques enables the advancement of evidence-based, effective strategies specifically tailored to the unique communication needs of individuals with RTT. Emerging technologies, including eye-tracking systems, brain–computer interfaces, and AI-supported AAC tools, have the potential to transform assessment and intervention frameworks. Although still underutilized in many clinical settings, these technologies offer promising avenues for accessing and interpreting communicative intent, particularly for individuals with limited expressive language.

While strategic planning remains an essential aspect of intervention, typically formulated before or during its implementation, the insights provided by this study offer valuable supplementary perspectives. These additional considerations contribute to a more comprehensive understanding of the communication-related challenges in RTT, informing practitioners and researchers as they strive to optimize intervention strategies.

A comprehensive understanding of communication in individuals with RTT must account for cultural, linguistic, and socioeconomic factors that shape access to services, caregiver involvement, and intervention outcomes. Inclusive methodologies are essential to ensure equitable support. Beyond initial assessment planning, families play a central role in daily implementation and emotional support, warranting greater emphasis. Effective intervention also requires a multidisciplinary approach, engaging professionals from various fields, including speech–language pathology, occupational therapy, psychology, education, and neurology, to meet diverse needs. Ethical dimensions, including the right to communication, informed consent for nonverbal individuals, and advocacy for inclusive services, must be actively integrated into both practice and research. This review highlights a pressing need for more rigorous, longitudinal studies that examine the trajectory of communication development across the lifespan in individuals with Rett syndrome (RTT). Future research should prioritize identifying predictors of communication success and intervention responsiveness, as well as conducting randomized controlled trials to rigorously evaluate the effectiveness of AAC, behavioral, and neuromodulatory interventions. There is a clear necessity to develop RTT-specific assessment instruments capable of capturing subtle communicative behaviors, including those conveyed through gaze or gestures, and to investigate how emerging technologies—such as AI-driven AAC systems and brain–computer interfaces—can be integrated into both assessment and intervention practices. Moreover, embracing cultural and linguistic diversity in study samples will be crucial to improving the global relevance and applicability of findings. A transparent and critical appraisal of the existing evidence base, combined with methodological innovation and inclusive research approaches, will be pivotal in shaping best practices and advancing equitable, person-centered communication support for individuals with RTT.

## 5. Conclusions

This narrative review integrated evidence from 57 studies published in the past two decades (2004–2024), providing a comprehensive overview of the verbal and nonverbal communication profiles of individuals with Rett syndrome (RTT). The findings indicate that, despite marked regression in verbal abilities common among this population, many individuals retain a repertoire of prelinguistic and nonverbal behaviors, such as eye-pointing, facial expressions, and gestural communication. When appropriately identified and supported, these preserved communicative behaviors can serve as functional and meaningful channels for expression, underscoring the importance of tailored intervention strategies to maximize communicative potential.

Regarding the assessment strategies, they frequently relied on several tools (e.g., Inventory of Potential Communicative Acts, Vineland Adaptive Behavior Scales, and eye tracking technology). However, there is a lack of standardized communication tools exclusively for individuals with RTT.

Assessment approaches frequently utilized instruments such as the Inventory of Potential Communicative Acts, the Vineland Adaptive Behavior Scales, and eye-tracking technology to evaluate communicative abilities in individuals with Rett syndrome. However, a persistent gap in the literature is the absence of standardized assessment tools specifically developed and validated for the RTT population, which limits the precision and consistency of communication profiling in both clinical and research contexts.

In relation to clinical practice, this narrative review highlights the importance of delivering interventions that are tailored to the individualized and responsive needs of each person with Rett syndrome, taking into account their distinct communicative and developmental profiles. It is recommended that professionals across various disciplines, including speech–language pathologists, occupational therapists, educators, and neurologists, collaborate with caregivers to implement personalized strategies. Crucially, active family involvement throughout the intervention process remains a central objective, as it contributes significantly to the consistency, relevance, and overall effectiveness of communication support.

In conclusion, future research should prioritize the development of standardized assessment instruments specifically designed for individuals with Rett syndrome, alongside rigorous investigations into the efficacy and applicability of technology-based intervention approaches.

## Data Availability

Data sharing is not applicable to this article.

## Appendix A

**Table A1:** Studies characteristics involved in this review.

					Results	
Authors	Sample [Age]	Purpose of the Study	Study Design	Communication Skills	Assessment of Communication Skills	Intervention of Communication Skills
*Cianfaglione* et al. [45]	42 individuals with RTT [4 to 47 years, and at follow-up was 7 to 48 years]	Gained a UK national sample of people with RTT across the age range and (1) conduct a cross-sectional comparison of age groups and (2) undertake a longitudinal follow-up.	Research study	Preverbal and pre-intentional level matches their cognitive skill bundle, inhibiting speech intelligibility, body and gestural engagements and expressing unwillingness and displeasure can often happen nonverbally, and receptive abilities are judged alongside mental age	VABS and RSBQ	NA
*Roche* et al. [15]	23 studies: 13 studies on ASD, 8 studies on RTT, and 2 studies on FXS [0–24 months]	Evaluated early vocal behaviors present in the first two years of life for individuals later diagnosed with ASD, RTT, or FXS.	Systematic analysis on the early development of vocalizations in children later diagnosed with ASD, RTT, or FXS	Inhibited learning ability, decreased mental lexicon development, and verbal communication skills being closely linked to RTTs’ cognitive skills	IPCA	NA
*Townend* et al. [19]	67 families who have individuals with RTT [3 years 6 months to 60 years 6 months]	A brief report on families’ experiences of eye gaze technology as one form of AAC for individuals with RTT and the advice, training, and support they receive about this.	Research study/online survey relating to communication skills and families’ experiences of communication support	Using eye gaze technology to express their communication abilities	Eye gaze technology	NA
*Sigafoos* et al. [30]	Eight studies that involved 41 individuals with RTT [2.7 years to 36 years]	Reviewed studies that aimed to determine whether behaviors, such as body movements, vocalizations, eye gaze, and facial expressions, served a communicative function for individuals with RTT.	Systematic analysis	Eye pointing/eye gaze, body movements, leading, clapping, reaching, pushing away items, tantrums/screaming	VABS, PPVT-3, Ordinal Scales of Psychological Development, behavioral observations, and interviews	NA
*Fabio* et al. [20]	20 children (10 individuals with RTT and 10 control girls, matched on mental age) [6 and 21 years]	Analyzed attention and communication abilities in RTT.	Research study	Joint attention, requesting, pointing at the object, and reaching for toys that were out of reach	VABS and behavioral observations	NA
*Elefant and Wigram* [39]	Seven girls with RTT [from 4 to 10 years]	Presented results of a research study examining learning ability in individuals with RTT.	Research study	Choice making and learning ability	NA	Choice-making through eye gaze, nose pointing, or with their hand, according to their preference song and ability
*Einspieler and Marschik* [8]	NA [infants during their first 2 months of life up to 3 years]	This review sheds light on atypical neurofunctions and potential behavioral biomarkers before the onset of the regression of RTT.	Review study	Eye contact, responsive smile, reaction to being called by name, cooing, vocalizations, babbling, index finger pointing, gestures, body movement, proto-words, and two-word combinations	NA	NA
*Segawa and Nomura* [42]	51 articles with individuals with RTT [NA]	NA	Review study	Avoidance of social interaction	NA	NA
*Urbanowicz* et al. [61]	16 parents who have individuals with RTT [from 2.3 to 33.7]	Investigated the communicative use of eye gaze and gestures in females with RTT.	Research study	Eye pointing, assigning a point to each feature, the ability to make choices, requesting attention, facial expressions, and social interactions	Interview/several questions about expressive and receptive communication	NA
*Fabio* et al. [22]	A girl with RTT [21 years old]	Investigated the possibility of training communication abilities in people with RTT.	Case study	Cognitive and communicative delays	NA	Training reading–writing abilities
*Wandin* et al. [44]	Nine individuals with RTT [15 to 52 years]	Aimed to (a) develop a tool for assessing visual attention in individuals with RTT using AAC with a communication partner during naturalistic interactions in clinical settings and (b) explored aspects of the tool’s reliability, validity, and utility.	Research study	Limited speech, no intelligible words, and word approximations used inconsistently	AVAI tool	NA
*Marschik* et al. [47]	An individual with RTT [The participant was longitudinally observed from birth to 11 years of age]	Assessed various aspects of speech and language and communicative functions of an individual with the preserved speech variant of RTT to describe her developmental profile over 11 years.	Case study	Hand stereotypies (such as rubbing and washing-like hand movements), as well as hand-to-mouth and hand-to-tongue stereotypies, morphosyntactic and socio-pragmatic limitations, and dysfluency of speech, caused mainly by immediate echolalia	Audio–video recordings of spontaneous speech, picture book reading, storytelling (five stories) and daily routines, parental diaries, and checklists: (i) the Austrian Rett survey; (ii) the Austrian adaptation of the MB-CDIs, a checklist to assess early socio-communicative functions, early gestures, vocabulary, and grammar; (iii) IPCA, AWST-R, TROG-D, SET-K, and the PADLD.	NA
*Ahonniska-Assa* et al. [9]	17 girls with RTT [3 years and 4 months to 12 years and 2 months]	Investigated whether eye-tracking technology in forced-choice tasks can enable children with very severe motor and speech impairments to respond consistently, allowing a more reliable evaluation of their language comprehension.	Research study	Inconsistent imitation and social skills	Eye-tracking technology and PPVT-4	NA
*Castelli* et al. [51]	Nine individuals with RTT [5 to 19 years old]	Investigated Theory of Mind capacities with RTT, which is similar to autism yet with more significant impairments.	Research study	Prelinguistic behaviors	Non-verbal false-belief task	NA
*Sigafoos* et al. [29]	Nine studies, 31 participants with RTT [2 years and 7 months to 17 years]	Reviewed communication intervention studies involving people with RTT.	Systematic analysis	Imitative speech, requesting, naming/commenting, and various receptive language skills	NA	Early intensive behavioral intervention
*Hagberg* [50]	NA	Reviewed presentation and clinical diagnosis of RTT at various ages and stages.	Review study	Eye communication or facial expressions as a prominent feature in most individuals with RTT	NA	NA
*Didden* et al. [34]	120 children and adults (girls) with RTT [5 to 55 years]	Assessed the forms and functions of prelinguistic communicative behaviors for 120 children and adults with RTT using the IPCA.	Research study	Eye gaze, laughing, smiling, vocalization, approaching a person, and distancing from a person	IPCA	NA
*Marschik* et al. [41]	Six individuals with RTT [aged 7 to 24 months]	Contributed new findings related to the pre-regression verbal development of females with a variant of RTT.	Research study	Babbling, (proto-)words and (proto-)word combinations, and nonverbal skills	Spontaneous speech analyses and additional parental report	NA
*Djukic* et al. [53]	37 Individuals with RTT and 34 typical development girls [2 to 31 years]	Examined the use of eye tracking technology, which is a technique uniquely suited for studying cognition in RTT.	Research study	Using eye gaze to communicate their desires, greet, point, request, and refuse	NA	NA
*Kaufmann* et al. [54]	80 individuals with RTT [1.6 to 14.9 years]	Examined the social impairments in RTT, the characteristics, and the relationship with clinical severity.	Research study	Pointing at or reaching for an object and requesting	SSI, a measure of autistic behavior suited for individuals with severe communication and motor impairment, RSBQ, VABS, and RSSS	NA
*Hetzroni* et al. [56]	Three individuals with RTT [8 to 10 years]	Investigated whether the use of assistive technology would assist in the ability to identify symbols of individuals with RTT.	Research study	Body movements for making choices, attention, and eye gaze movement	Observations of classroom activities and informal teacher and parent interviews	NA
*Marschik* et al. [21]	15 young individuals with RTT [first 2 years]	Aimed at shedding light on pre-regression development in RTT by focusing on early speech and language development using a video database.	Research study	Cooing, babbling, (proto-)words, word combinations and vocalization atypicalities	NA	NA
*Fabio* et al. [59]	28 Participants with RTT [4 to 22 years]	Examined the effects of cognitive rehabilitation with eye-tracker technology on attention, choice behaviors, and language over 2 years in patients with RTT.	Research study	Eye pointing, choice making, gestures, and body movements	NA	Cognitive rehabilitation
*Skotko* et al. [60]	Four individuals with RTT and their mothers [3.6 to 7 years old]	Described evidence and intervention strategies for parents, educators, and researchers who seek to enhance communication and literacy in individuals with RTT.	Research study	Attention and vocalizations	NA	Parent–child storybook interactions
*Smeets* et al. [52]	Two girls with RTT [9 and 11.5 years]	Discussed the clinical aspects with special emphasis on the behavioral phenotype and reviewed current perspectives in clinical management alongside perspectives on altering gene expression.	Review study	Dyspraxia, attention, and eye pointing	NA	NA
*Barnes* et al. [63]	74 individuals with RTT [2 and 11 years]	Examined the profiles of anxious behavior in individuals with RTT, with a focus on identifying the instrument with the best psychometric properties in this population.	Research study	NA	RSBQ, ADAMS, ABC-C, VABS-II, and CHQ	NA
*Byiers and Symons* [62]	NA [NA]	NA	Commentary study	NA	Eye-tracking technology	NA
*Cass* et al. [58]	87 individuals with RTT [2 years 1 month to 44 years 10 months]	Presented systematic data from a multidisciplinary clinical assessment of a large series of females with RTT.	Research study	Eye pointing, understanding cause and effect, making choices, and using words with and without meaning	NA	NA
*Sigafoos* et al. [13]	16 studies that involved 100 participants with RTT [NA]	Identified and summarized 16 communication intervention studies for individuals with RTT.	Systematic–narrative review	NA	NA	AAC device, graphic symbols, or activated microswitches/speech-generating devices to request preferred items, music therapy, eye tracking technology, and transcranial stimulation
*Lotan and Ben-Zeev* [33]	NA [NA]	Presented the basic understanding of common characteristics typical of this disorder and the variants from the classical expression of RTT.	Review study	Express a few words or partial vocabulary	NA	AAC
*Koppenhaver* et al. [75]	6 individuals with RTT [6 to 7 years old]	Explored the impact of resting hand splints, light-tech augmentative communication systems, such as voice-output devices and symbols, and very basic parent training on the symbolic communication and labelling behaviors of six individuals with RTT.	Research study	NA	NA	Picture communication symbols, single-message Big-Mack, a multi-message four in-line cheap talk, a variety of stands made from PVC pipe
*Ibrahim* et al. [86]	Five children with speech and physical impairments [6 to 9 years]	Examined how children and school staff interact in task-oriented events when speech SGDs are not present or focal.	Research study	NA	NA	AAC
*Fabio* et al. [64]	Three individuals with RTT [aged 29, 30, and 31 years old]	Examined the neurophysiological and cognitive effects of tDCS in three individuals with RTT with chronic language impairments.	Research study	NA	VABS and RARS	tDCS and cognitive empowerment
*Lamb* et al. [70]	396 caregivers who have individuals with RTT [1.6 to 50 years]	Investigated factors related to family functioning and adaptation in caregivers of individuals with RTT.	Research study	NA	PSOC, WCC-R, FAM-III, and PAS	PMT and CET intervention
*Byiers* et al. [71]	Three individuals with RTT [ages 15 to 47 years]	Examined the effectiveness of functional assessment and functional communication training methods for teaching three children with classic RTT novel communicative behaviors.	Research study	NA	Functional assessment	Functional communication training
*Stahlhut* et al. [74]	40 participants (parents, care assistants, professionals from schools, and professionals at day centers of RTT) [NA]	Explored facilitators and barriers to “uptime” (non-sedentary) activities in Danish girls and women with RTT as perceived by parents and professionals using focus groups.	Research study	NA	NA	Eye gaze computer
*Lim* et al. [72]	62 articles with communication skills of children’ RTT [from 9 to 60 years]	Summarized existing interventions and their outcomes in RTT rehabilitation and identified gaps in the literature.	Scoping review	NA	NA	Communication interventions to improve choice-making, communicative language, and social communication abilities
*Wandin, Lindberg and Sonnander* [87]	Three women with RTT [aged 27, 29, and 31 years]	Examined the effect of a communication intervention package on expressive communication and visual attention in individuals with RTT.	Case studies	Visual attention	NA	ALM using responsive partner strategies and a gaze-controlled device
*Wandin* et al., 2015 [73]	320 SLPs of RTT [NA]	Investigated communication intervention that SLPs provide to people with RTT.	Research study	NA	NA	AAC and communication aids
*Loffler and Gordon* [80]	NA [NA]	NA	Review study	NA	NA	Eye-tracking technology
*Sandberg* et al. [40]	Eight individuals with RTT [11 to 36 years]	Investigated the interrelationships between communication, cognition, and autistic features in young women with one of two variants of RTT complex disorders.	Research study	Loss and reappearance of verbal speech abilities, joint attention, and intentional communication	Video recordings, observations, and VABS	NA
*Kolb* et al. [69]	Three individuals with RTT [1.75 to 29 years old]	Systematically replicated the training procedures to teach three individuals with RTT to use AAC to make requests through caregiver coaching by researchers via telehealth.	Research study	NA	SDA	AAC in the form of manually activated single-message microswitches, eye-gaze device, mind training
*Neul* et al. [88]	925 caregivers who have individuals with RTT [From less than 1 year to over 40 years]	Determined the top concerns in RTT and RTT-related disorders.	Research study	Communication difficulties and physical inability to coordinate hand movements for communication	CGI-S and the RTT CSS	NA
*Percy* et al. [57]	NA [from 2 to 47 years for clinical studies using the Rett Syndrome Behavior Questionnaire, and the phase 3 LAVENDER study, aged 5 to 20 years]	Reviewed the RSBQ and its utilization in the assessment of symptoms associated with RTT.	Review study	Eye pointing and vocalizations	RSBQ	Eye gaze device, switches, and iPad
*Vilvarajan* et al. [48]	103 girls with RTT [aged less than 18 years]	Comprehensive review of the clinical features, comorbidities, and multidisciplinary management of a well-characterized cohort of females with classical RTT.	Research study	Single words or short phrases, body language, eye gaze, gestures, and vocalizations	NA	NA
*Romano, Lotan and Fabio* [49]	195 girls and women with RTT [3 to 40 years]	Evaluated the severity level of girls and women with RTT living in Italy and Israel, two countries with different approaches to caring for people with complex disabilities.	Research study	Spatial and temporal orientation, memory, verbal communication skills, non-verbal communication through facial expressions, ability to maintain eye contact and shared attention, and the presence of responsive smiling	RARS	NA
*Portnova* et al. [65]	32 Individuals with RTT and 41 typically developing girls [1.9 to 17.1 and 2.58 to 17.98]	Combined clinical, qualitative, and experimental/quantitative approaches to the resting EEG analysis in a search for the EEG characteristics that are linked with RTT symptoms.	Research study	Pleasure and displeasure sounds, and representational gestures	VABS, PDMS-2, and PLS-5	NA
*Fabio* et al. [55]	28 young and women with RTT [4 to 22 years]	Examined the effects of motor training on attention, reaching skills, and stereotypies in patients with RTT.	Research study	Attention, reaching skills, and stereotypies	VABS and RARS	Intervention in motor abilities and cognitive intervention
*Wandin, Lindberg and Sonnander* [46]	Three adults with RTT [27, 29 and 31 years old]	Explored and describe a trained communication partner’s use of responsive strategies in dyadic interaction with adults with RTT.	Research study	Eye gaze, body movements, and simple hand movements	RAACS	Gaze-controlled device
*Unholz-Bowden* et al. [66]	Three individuals with RTT [3, 5 and 19 years old]	Investigated the use of high- and low-tech AAC modalities by three individuals with RTT given similar instruction for using both modalities.	Research study	Gestures, requesting, pointing, and eye gaze	NA	High- and low-tech AAC instruments
*Girtler* et al. [89]	Three individuals with RTT [3, 5 and 19 years old]	Evaluated the effects of systematic individualized instruction procedures on the page-linking skills of individuals with RTT.	Research study	Gestures, laughing, crying, and vocalizations	IPCA and SDA	AAC
*Xavier* et al. [90]	14 girls with RTT and 11 girls with typical development [7–13 years and 3–4 years]	Evaluated the receptive vocabulary of individuals with RTT using eye-tracking technology and examined how these objective measures compared with parents’ perceptions of their daughters’ language abilities.	Research study	NA	Kerr Scale, a parental questionnaire on communication skills, the Peabody Picture Vocabulary Test (PPVT-4), and eye-tracking equipment	NA
*Passaro* et al. [91]	A narrative review NA [NA]	Evaluated studies conducted on the use of ETT to improve cognitive abilities in girls with RTT and to examine its potential application.	Review	NA	Eye tracking technology	NA
*Neul* et al. [67]	187 females with RTT [5 to 20 years old]	Evaluated the efficacy of trofinetide in communication of individuals with RTT.	Research study	Inability to communicate or make choices	The caregiver-rated CSBS-DP-IT social composite score and RTT-specific clinician rating scales related to discrete aspects of communication (RTT-COMC and RTT-VCOM)	NA
*Lee* et al., 2024 [28]	A case study with a female and a Narrative review with RTT [22 years old]	Evaluated the impact of AAC devices on communication outcomes and quality of life in individuals with RTT through a case report and narrative review.	Research study	NA	AAC	AAC
*Voniati* et al., 2024 [92]	Scoping Review with 7 articles with a total of 381 participants with RTT [NA]	Aimed to provide an overview of the identification of communication abilities in children with RTT in their daily routine from the perspectives of communication partners who interact with individuals diagnosed with RTT.	Scoping review	Eye gaze, expressing pleasure, requesting desired items, making choices, vocalizations, pointing, interaction, and strong receptive skills	Functional Ability Checklist, Functional Independence Measure for Children Communication and Symbolic Behavior Scales Developmental Profile Infant–Toddler Checklist, MacArthur–Bates Communicative Development Inventories and sections of the Vineland Adaptive Behavior Scales, Second Edition, the Assessment of Visual Attention in Interaction tool, the Child Participation in Family Activities questionnaire, the Clinical Severity Score and Hoffer Ambulation Scale	NA
*Voniati* et al., 2023 [43]	Narrative Review: 19 articles with individuals with RTT [NA]	Aimed to support the clinical work of speech–language pathologists (SLPs) while assessing the communication aptitudes of children with RTT.	Review	NA	Medical and developmental intake, functional assessment, Mullen scales, IPCA, PPVT, Vineland Adaptive Behavior Scales, a communication matrix, and AAC assessment	NA

RTT: individuals with Rett syndrome, ASD: Autism Spectrum Disorder, NA: non-applicable, FXS: fragile X syndrome, VABS: Vineland Adaptive Behavior Scale, RSBQ: Rett Syndrome Behavioral Questionnaire, PDMS-2: Peabody Developmental Motor Scale 2, IPCA: Inventory of Potential Communicative Acts, AVAI Tool: Assess Visual Attention in Rett syndrome, PSOC: Parenting Sense of Competence Scale, WCC-R: ways of coping checklist, revised, FAM-III: Family Assessment Measure III, PAS: psychological adaptation scale, tDCS: Transcranial Direct Current Stimulation, MB-CDIs: The MacArthur–Bates Communicative Development Inventories, AWST-R: Aktiver Wortschatztest für 3- bis 5-jährige Kinder–Revision–(German vocabulary Test), TROG-D: Test zur Überprüfung des Grammatikverständnisses (Test for the Reception of Grammar), SET-K: SprachEntwicklungsTest für Kinder (speech and language development test), PADLD: Patholinguistic Assessment of Developmental Language Disorders, SSI: Screen for Social Interaction, ADAMS: Anxiety, Depression, and Mood Scale, ABC-C: Aberrant Behavior Checklist-Community, CHQ: child health questionnaire, PMT: parent management training, CET: coping effectiveness training, SGDs: speech generating devices, RARS: Rett Assessment Rating Scale, RAACS: Responsive Augmentative and Alternative Communication Style Scale, PLS-5: preschool language scale, CGIS-S: Clinical Global Impression-Severity, CSS: clinical severity score, SDA: structural descriptive assessment, ALM: aided language modelling, AAC: Augmentative and Alternative Communication, EEG: Electroencephalography.

## Appendix B

**Table A2:** SANRA Brief Scale for the quality assessment of the current narrative review.

SANRA Criterion	Score (0–2)	Justification for Scoring
1. Importance for the readership	2	The introduction contains all the elements to explain the significance of the communication deficits of individuals with RTT.
2. Stated aims or research questions	2	The aims of the study are stated clearly in the abstract and Section 1.2 with main research questions and subobjectives.
3. Description of the literature search	2	A transparent, structured search is described. The databases, keywords, Boolean, date range, inclusion/exclusion criteria, and the use of Kable’s 12-step method are noted.
4. Referencing	2	Up-to-date, relevant, and peer-reviewed references (*n* = 91) were used from Databases.
5. Scientific reasoning	2	The discussion was developed by making a synthesis of the findings across all studies and discussed supported with citations and any possible evidence.
6. Appropriate presentation of data	2	The results were described by domain (verbal, nonverbal, assessment, intervention) and presented by a table in the Appendix A.
Total Score	12	
